# Analysis and characteristics of coronaridine, an alkaloid found in *Catharanthus roseus*

**DOI:** 10.5511/plantbiotechnology.24.0717a

**Published:** 2024-12-25

**Authors:** Hiroaki Kisaka, Sachise Karakawa, Tetsuya Miwa, Hiroto Hirano, Takashi Onuki, Mayu Iyo

**Affiliations:** 1 Research Institute for Bioscience Products & Fine Chemicals, Ajinomoto Co., Inc., 1-1, Suzuki-cho, Kawasaki-ku, Kawasaki, Kanagawa 210-8681, Japan

**Keywords:** alkaloid, *Catharanthus roseus*, coronaridine, high temperature, LC-MS/MS

## Abstract

Coronaridine, a monoterpenoid indole alkaloid, is present in *Tabernanthe iboga* and the related species *Tabernaemontana divaricata*. Recent exhaustive analysis revealed its presence in *Catharanthus roseus*, though specific details remain unknown. We conducted a detailed analysis of coronaridine in *C. roseus*, detecting it in seedlings post-germination up to 8 weeks after sowing, with peak abundance at 3–4 weeks. Gradual decrease occurred from the flowering stage, and it was absent during seed formation. The accumulation varied dramatically with the plant’s growth phase. LC-MS/MS analysis confirmed (−) coronaridine, consistent with *T. iboga*. Additionally, cultivating at 35°C increased coronaridine accumulation over 10-fold. These findings hold potential for enhancing the stable production of iboga alkaloids for pharmaceutical use.

## Introduction

Various species within the *Apocynaceae* family, such as *Tabernanthe iboga*, *Voacanga africana*, and numerous *Tabernaemontana* species, are recognized for producing iboga-type alkaloids. Ibogaine, an iboga-type alkaloids, has shown promise as a medicinal drug. Several studies, both in animals and in humans, report long-term drug abstinence from various substances, including opioids, alcohol, and psychostimulants, and sustained reductions in depressive symptoms after ibogaine administration (Brown and Alper 2018; Mash 2018; Mash et al. 2000, 2018; Santos et al. 2017; Schenberg et al. 2014; Wasko et al. 2018). *T. iboga* is a plant native to Africa and is a limited resource. This fact making it difficult to ensure a stable supply as a pharmaceutical ingredient.

**Figure 1:**
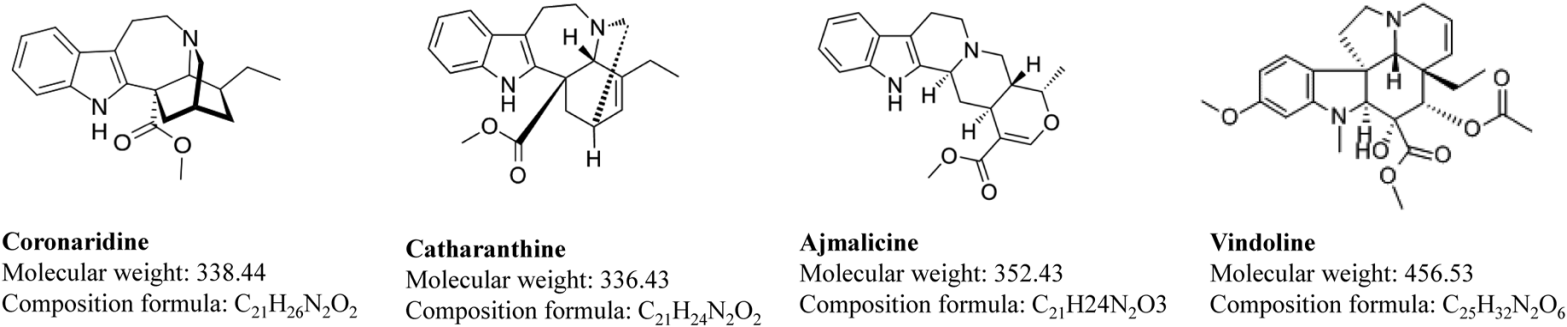
Figure 1. Structural formula, molecular weight, and composition formula of coronaridine, catharanthine, ajmalicine and vindoline appearing in this study.

Farrow et al. (2018) examined iboga alkaloid metabolism in *Tabernanthe iboga*, revealing coronaridine as precursors. Their study reported two enzymes, cytochrome P450 (I10H) and *O*-methyltransferase (N10OMT), essential for iboga alkaloid biosynthesis from coronaridine. Coronaridine (Figure 1), also known as 18-carbomethoxyibogamine, is an alkaloid found in *Tabernanthe iboga* and related species, including *Tabernaemontana divaricata* (Singh et al. 2011).

*Catharanthus roseus*, also a member of the *Apocynaceae* family, is a widely cultivated flowering plant known for generating approximately 130 different alkaloids. Among these, vincristine and vinblastine are prominent compounds used to treat leukemia and Hodgkin’s lymphoma (Dhayanithy et al. 2019). *C. roseus* also contains relatively large amounts of alkaloids such as catharanthine, vindoline, and ajmalicine (Figure 1), and catharanthine is classified as an iboga-type alkaloid. Recent gas chromatography-mass spectrometry (GC-MS) analysis has identified the presence of coronaridine in *C. roseus* (Bansal et al. 2023; Rani et al. 2021). If *C. roseus* contains large amounts of coronaridine, it may be possible to semisynthetically produce iboga alkaloids using that coronaridine. Therefore, we focused on and characterized the coronaridine found in *C. roseus*.

## Materials and methods

### Cultivation and sampling of *C. roseus* plants

The seeds of *C. roseus* used were Pacifica XP cv Really Red (Takii Seed Co., Ltd.). Two seeds were sown in watered Jiffy Seven (Sakata Seed Corporation). Cultivation occurred at 25°C temperature, 60% humidity, and 100–150 μmol m^−2^ s^−1^ light amount. After one week, one fully germinated plant was retained, and another was removed. Following another week of cultivation, plants were transferred to 6-cm pots with supplemented culture soil. Otsuka Liquid Fertilizer (OAT Agrio Co., Ltd.) at a 2,000-fold dilution was used for weekly plant fertilization.

Plants cultivated at different temperatures were transplanted into soil and grown at either 25°C or 35°C for 3 weeks. Three plants with uniform growth were selected, and 0.2 g of leaves were sampled from the second and third leaves from the growth point. To confirm the difference in the amount of coronaridine accumulated in young and old leaves, we used three plants grown at 25°C for 8 weeks after sowing. 0.2 g of the 2nd to 3rd leaf from the growth point or the 4th to 5th leaf from the base was sampled. The plants sown weekly were transplanted to 35°C one week after sowing, and one week later, they were planted in soil and continued cultivation at 35°C. Three independent plants were prepared for each week-old plot, and 0.2 g of each plant was sampled using the 2nd to 4th leaves from the growth point. A 3-mm diameter zirconia bead was placed in a 2-ml microtube (Eppendorf Co., Ltd.), and 0.2 g of measured leaf material was added. The samples were frozen using liquid nitrogen, and stored at −80°C until all samples were collected.

### Alkaloid extraction

A frozen sample was crushed with MM400 (Retsch), and 1 ml of methanol was added to a 2-ml microtube. Using a microtube mixer (Tokyo Rikakikai), the samples were mixed at 2,000 rpm and 28°C for 2 h. Then, centrifugation was performed at 25°C at 14,000×g for 20 min, and the supernatant was filtered using an Ultra-free-MC 0.22-µm filter unit (Millipore). The filtrate was used for analysis.

### Solid-phase extraction conditions

A mobile phase A solution was prepared using 10 mM ammonium acetate, 0.1% acetic acid, and water. The methanol extract of the sample (200 µl) was mixed with 3,800 µl of mobile phase A. Oasis HLB 3 ml (60 mg) Extraction cartridges (Waters) served as the solid-phase extraction column. Column conditioning included 2 ml of methanol, 1 ml of water, and 2 ml of mobile phase A. Loading the column with 4 ml of the sample was followed by a wash with 3 ml of 70% methanol in water. Elution utilized 1.5 ml of 100% methanol, and the eluate was dried under reduced pressure at 45°C for 1 h. After adding and dissolving 60 µl of methanol, 40 µl of water was added and mixed to prepare the analysis sample.

### LC-MS and LC-MS/MS measurements

We were kindly provided with standard coronaridine by professor Hirasawa of Hoshi University. They conducted research on the isolation and usefulness of monoterpenoid indole alkaloid from *Tabernaemontana divaricata*, and coronaridine was one of these alkaloids (Hirasawa et al. 2021). Coronaridine was analyzed by LC-MS using a Nexera X2 HPLC system from Shimadzu (Kyoto, Japan) and Q-Exactive™ puls from Thermo Fisher Scientific (Sunnyvale, CA, USA). A Waters Acquity UPLC® BEH C18 (2.1×100 mm, 1.7 µm) column was used for analysis. Mobile phase A: 10 mM ammonium acetate/0.1% acetic acid/water; mobile phase B: methanol; flow rate: 1 ml min^−1^; column temperature: 40°C; and gradient conditions: 0–30 min from 60% to 95% mobile phase B, 30–40 min maintained at 95%, 40–40.1 min from 95% to 60%, and 40.1–50 min at 60% for reequilibration. The injection volume was 10 µl PDA detection was performed at 190–400 nm.

In the MS system, full mass scan measurement and PRM measurement (MS/MS of target analyte) were performed. The full mass scan parameters were set as follows: polarity, positive; scan range, 100–1,000 *m*/*z*; resolution, 70,000; AGC target, 3×e^6^; and maximum IT, 200 ms. The PRM parameters were set as follows: resolution, 17,500; default charge state, 1; AGC target, 2×e^5^; maximum IT, 100 ms; isolation window, 4.0 *m*/*z*. The collision energy (CE) was set at 30, 50, and 60 V.

### LC-CD measurement

For LC-CD analysis, a JascoX-LC system (JASCO Corporation) and CD-2095 Plus detector (JASCO Corporation) were used. The column was Intertsil ODS-3 (4.6×250 nm, 5 µm) (GL Science, Tokyo, Japan), the column temperature was 40°C, mobile phase A was 10 mM ammonium acetate/0.1% acetic acid/water, and mobile phase B was methanol. Flow rate: 1 ml min^−1^; injection volume: 10 µl. The gradient conditions were as follows: 0–30 min from 60% to 95% mobile phase B, 30–40 min maintained at 95%, 40–40.1 min from 95% to 60%, and 40.1–50 min at 60% for reequilibration. PDA detection was performed at 190–400 nm. CD detection parameters were as follows: wavelength, 280 nm; response, STD; polarity, positive; gain, ×10; UV range, short; threshold, 0.01 AU; CD range, 0.005°; scan speed, STD.

## Results

### LC-CD, LC-MS, and LC-MS/MS analysis

**Figure 2:**
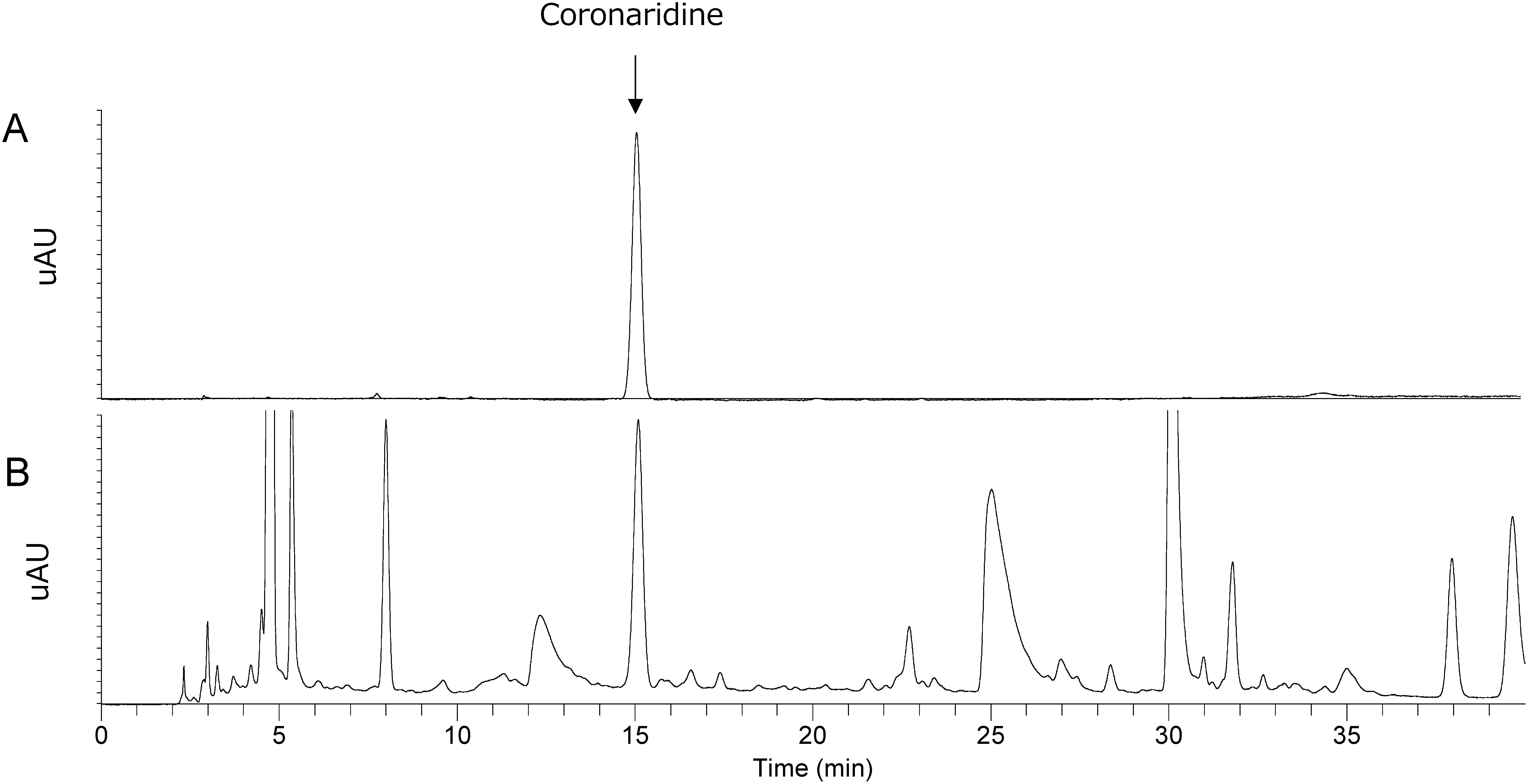
Figure 2. LC-MS measurement. A, standard coronaridine 10 µg ml^−1^. B, *C. roseus* solid-phase extract. Arrow indicates coronaridine.

**Figure 3:**
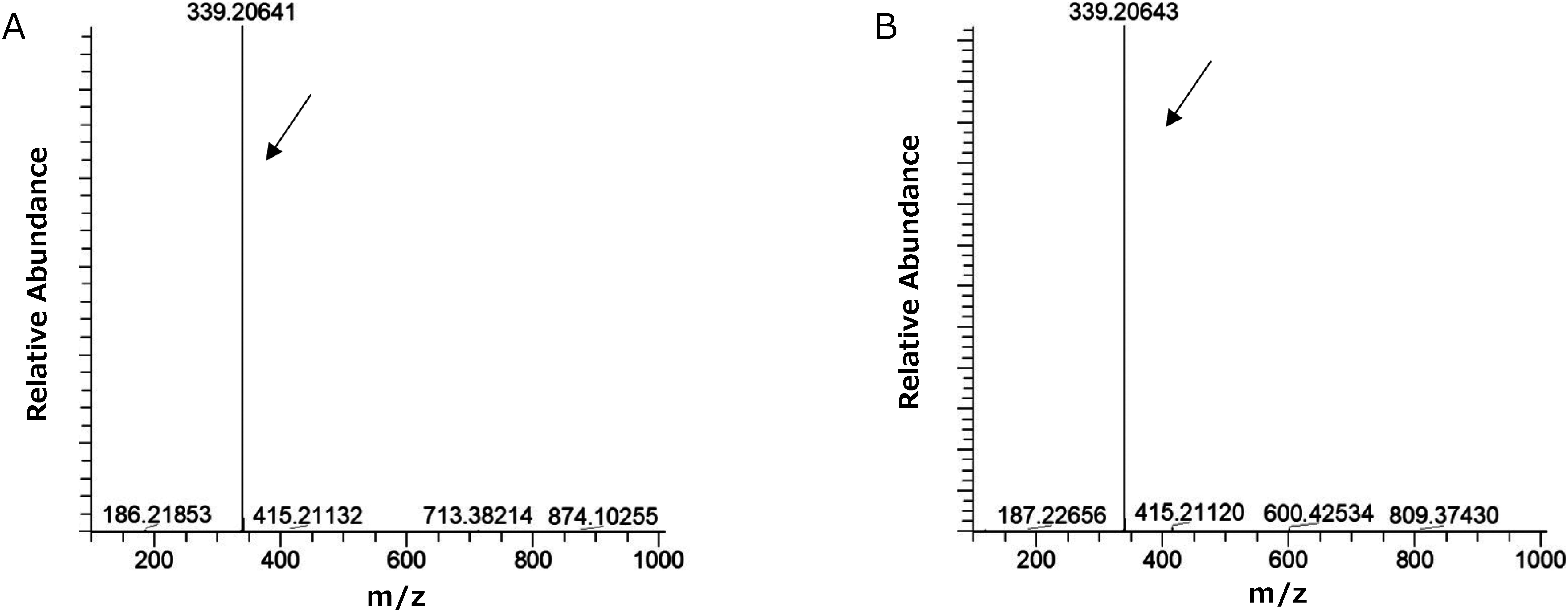
Figure 3. LC-MS measurement. MS spectrum. A, standard coronaridine 10 µg ml^−1^. B, *C. roseus* solid-phase extract (Retention time, 15 min). Arrow indicates coronaridine.

**Figure 4:**
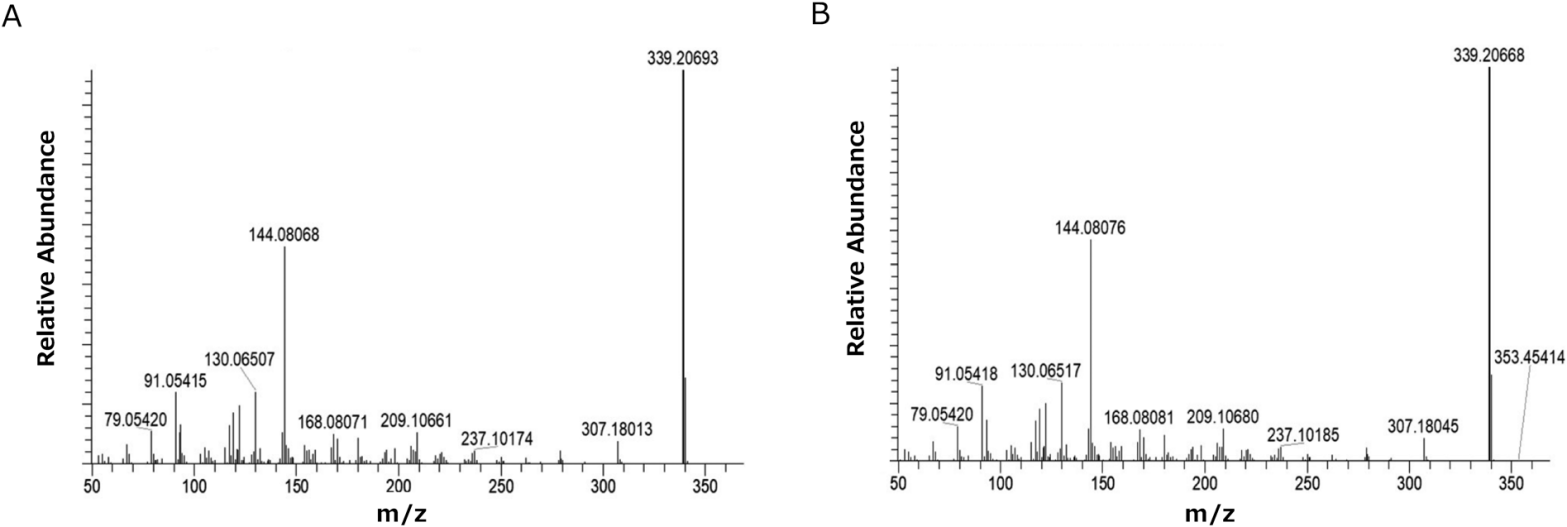
Figure 4. LC-MS/MS measurement. MS/MS spectrum of peak detected at retention time 15 min of *C. roseus* extract. A, standard coronaridine 10 µg ml^−1^. B, *C. roseus* solid-phase extract (Retention time, 15 min).

In LC-MS analysis, a peak corresponding to standard coronaridine at 15 min retention time was observed in the solid-phase extract of *C. roseus* (Figure 2). Although solid phase extraction was performed, alkaloids (e.g. vindoline and ajmalicine) with hydrophobicity equivalent to coronaridine were also detected. Further comparison of extracted ion chromatograms (*m*/*z* 339.20–339.21) revealed matching peaks at RT15 min for standard coronaridine and the *C. roseus* extract (Figure 3). No ions from other components were detected, confirming coronaridine as the main component of the RT15 min peak. LC-MS/MS analysis with CE values of 30, 50, and 60 confirmed that the MS/MS spectra of the *C. roseus* extract at RT15 min matched the standard coronaridine spectrum (Figure 4). Furthermore, it was estimated from accurate mass spectrometry that *m*/*z* 339.20643, estimated composition formula C_21_H_27_O_2_N_2_. These results suggested the presence of coronaridine in the leaves of *C. roseus*.

**Figure 5:**
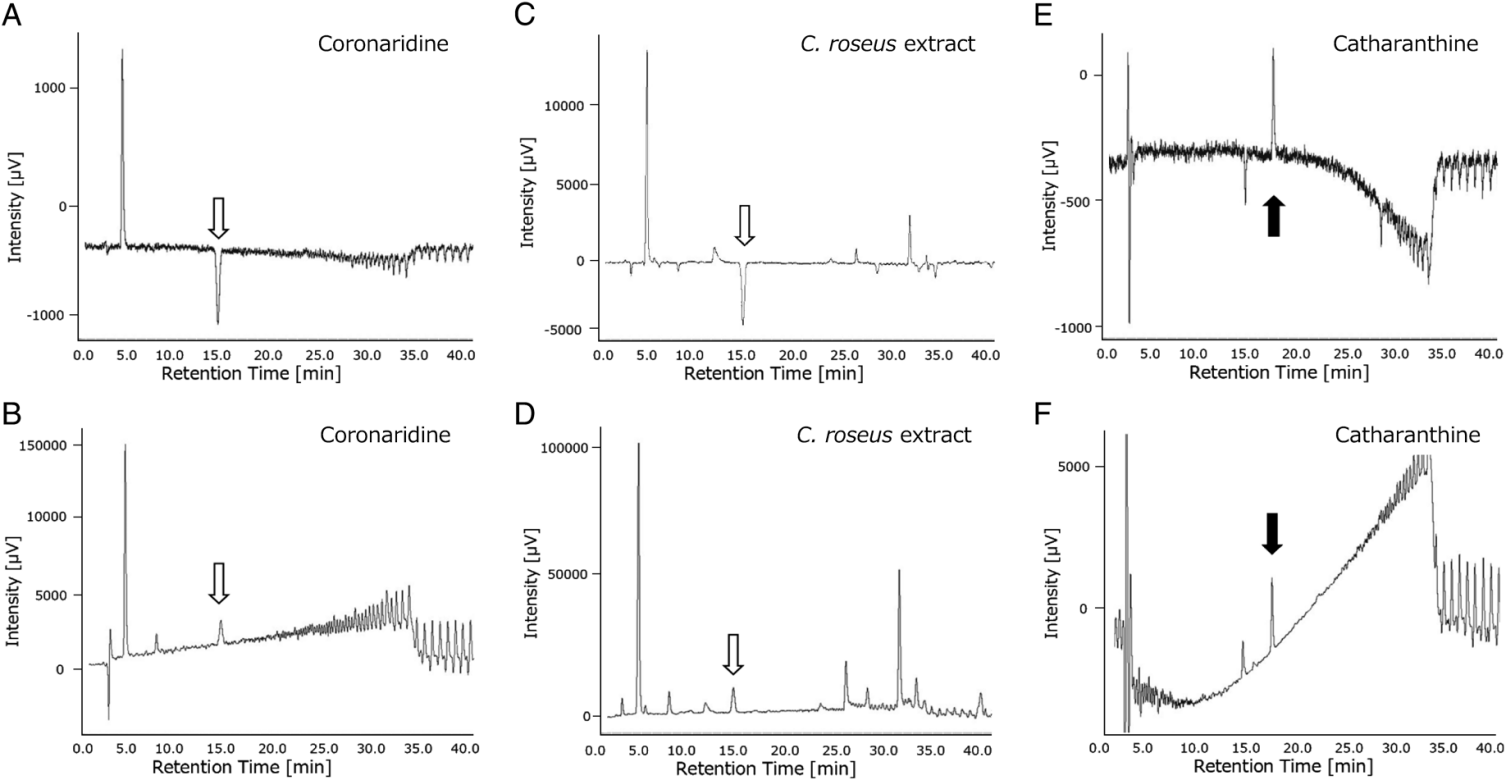
Figure 5. LC-CD measurement. A and B, standard coronaridine 10 µg ml^−1^. C and D, *C. roseus* solid phase extract. E and F, standard catharanthine 50 µg ml^−1^. A, C and E, CD chromatogram (280 nm). B, D and F, UV chromatogram (280 nm). White arrows indicate coronaridine. Black arrows indicate catharanthine.

**Figure 6:**
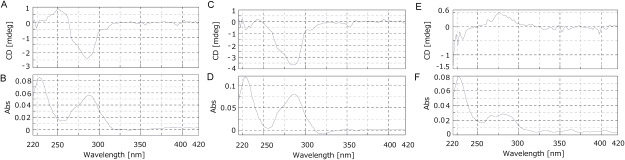
Figure 6. CD measurement. A and B, standard coronaridine 100 µg ml^−1^. C and D, *C. roseus* solid-phase extract (Retention time 14 min). E and F, standard catharanthine 100 µg ml^−1^. A, C and E, CD chromatogram. B, D and F, UV chromatogram. Abs: Absorbance.

Methanol extraction of *C. roseus* leaves six weeks after sowing, followed by LC-CD analysis, revealed a negative peak in the *C. roseus* extract at the same retention time (RT14 min) as standard coronaridine (Figure 5). Additionally, CD spectrum measurement indicated matching CD spectra between standard coronaridine and the RT14 min peak of the *C. roseus* extract (Figure 6A). Furthermore, as a control, we measured the CD of catharanthine, which is known to have a (+) CD spectrum (Figure 6E), and the CD spectrum was detected in the opposite direction to that of coronaridine extracted from *C. roseus* (Figure 6C). From the above results, coronaridine contained in *C. roseus* was estimated to be (−) coronaridine.

### Improved accumulation by high-temperature treatment

**Figure 7:**
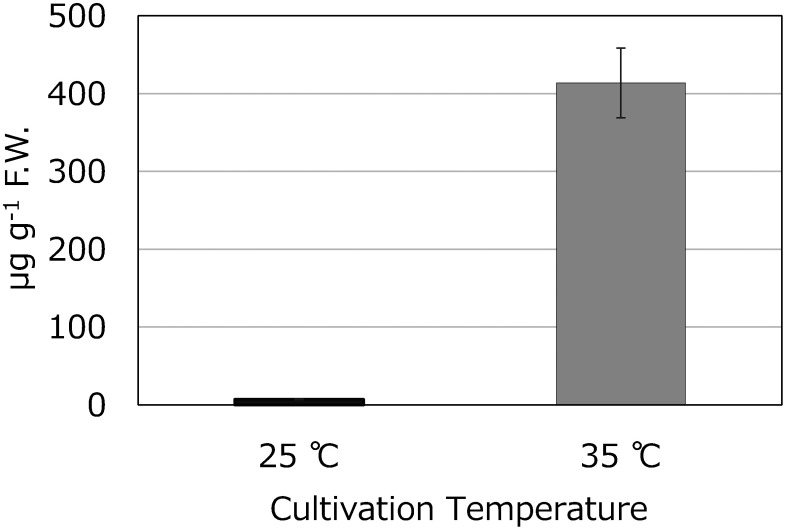
Figure 7. Coronaridine content in leaves of *C. roseus* 4 weeks after sowing grown at 25°C or 35°C (*n*=3).

**Figure 8:**
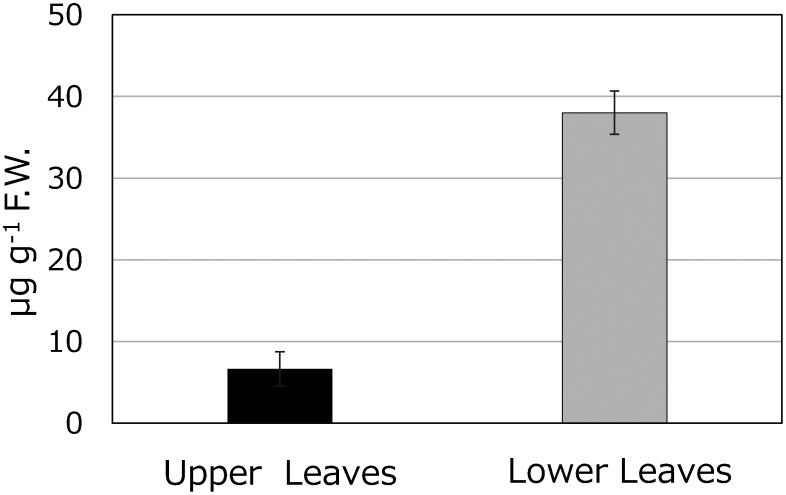
Figure 8. Coronaridine content in upper leaves and lower leaves of *C. roseus* 8 weeks after sowing grown at 25°C (*n*=3).

Typically, *C. roseus* is cultivated within a temperature range of 20°C to 30°C, with 25°C considered optimal. In this experiment, the *C. roseus* under study was cultivated at the standard temperature of 25°C. While investigating cultivation conditions to enhance coronaridine content during early growth stages, it was observed that cultivating at a temperature of 35°C increased coronaridine levels in leaves. At 4 weeks of age, leaves from plants grown at 25°C contained 30 mg g^−1^ F.W. of coronaridine, while those from plants grown at 35°C contained a significantly higher 320 mg g^−1^ F.W. of coronaridine (Figure 7). Furthermore, a comparison of coronaridine content between upper and lower leaves of a single plant revealed that lower leaves had a higher content, measuring 38.0 µg g^−1^ F.W., compared to 6.7 µg g^−1^ F.W. in upper leaves (Figure 8).

**Figure 9:**
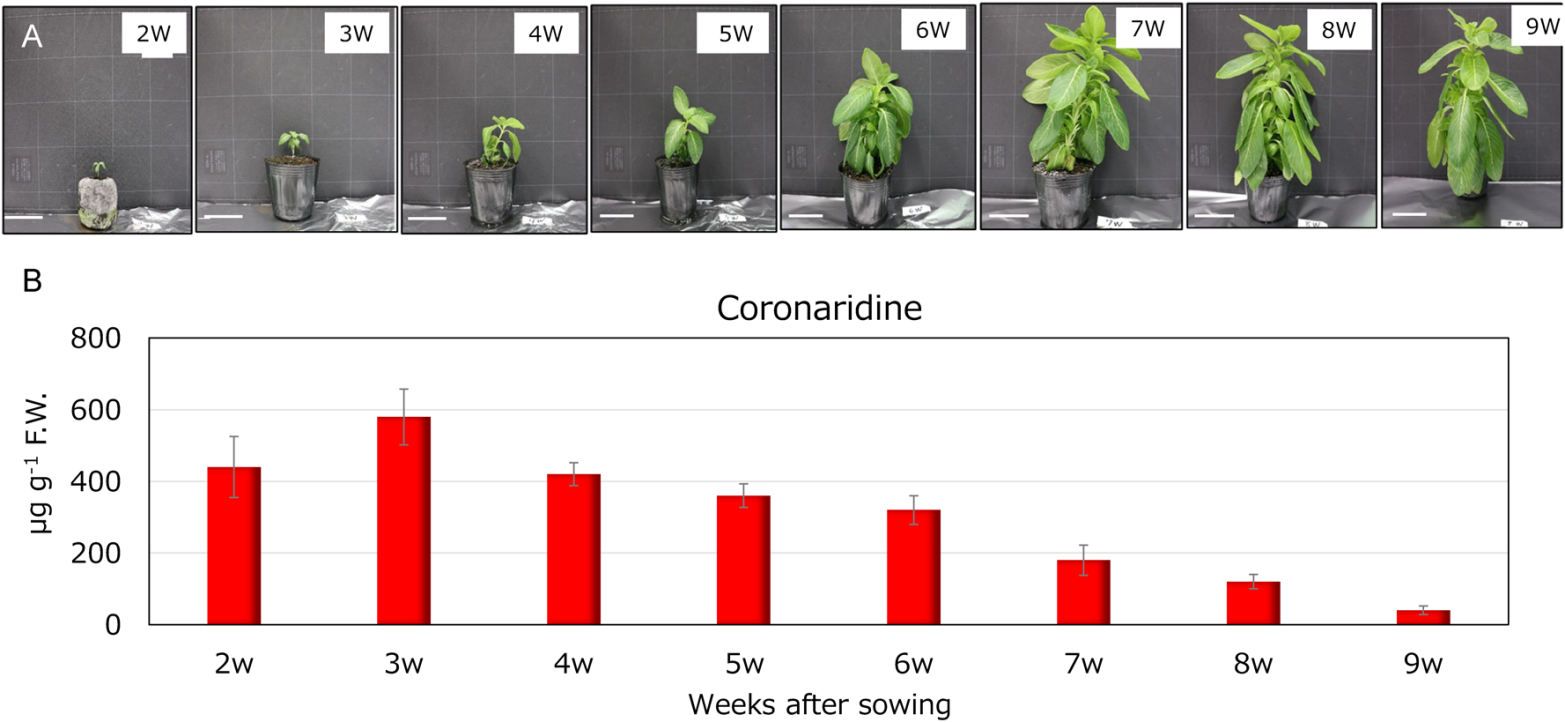
Figure 9. Coronaridine content of *C. roseus* leaves from 2 to 9 weeks after sowing grown at 35°C. A, plant growth at each growth stage. B, coronaridine content in plant leaves at each stage (*n*=3). Bar: 5 cm.

**Figure 10:**
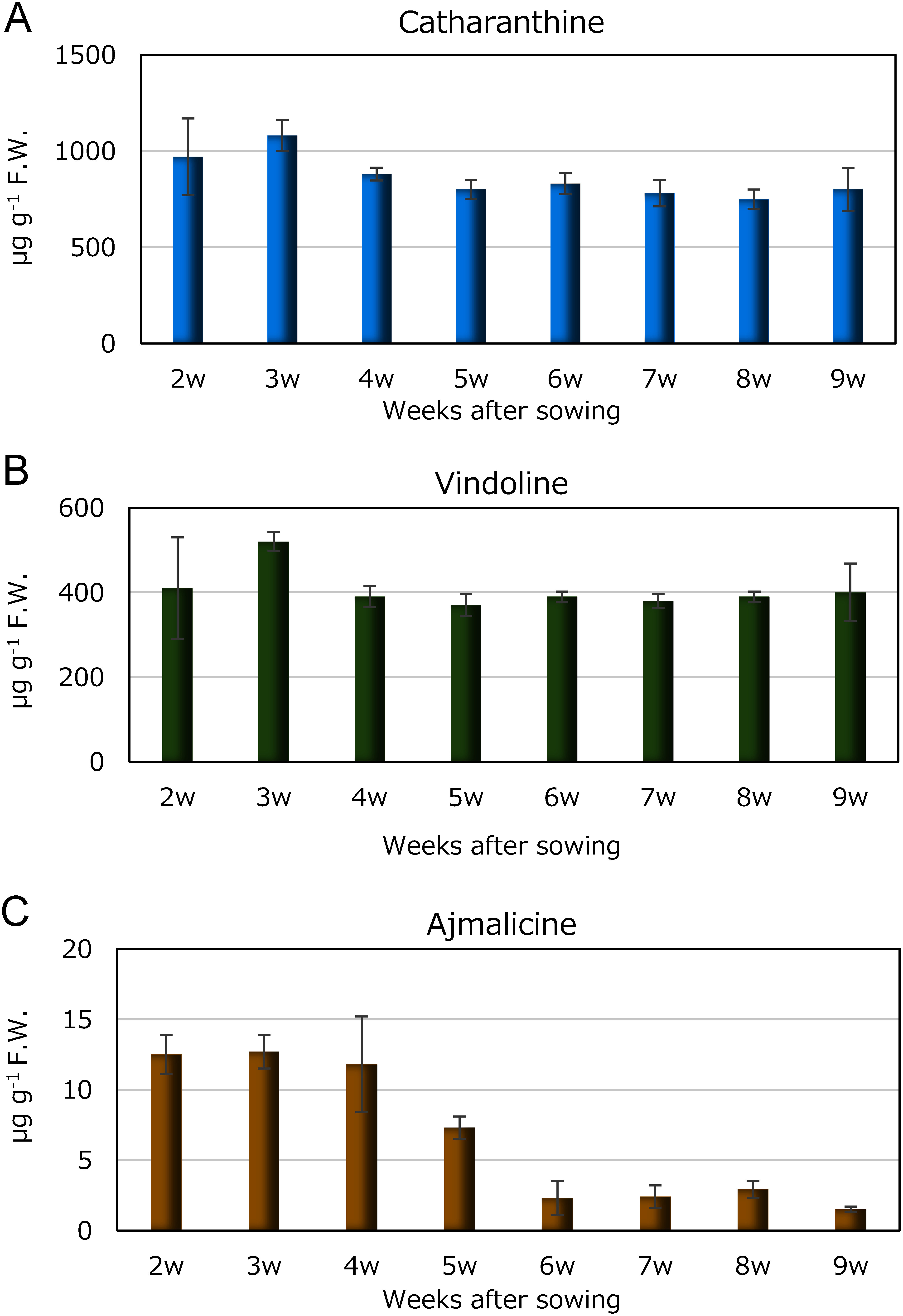
Figure 10. Contents of catharanthine, vindoline, ajmalicine in *C. roseus* leaves from 2 to 9 weeks after sowing grown at 35°C (*n*=3). A, catharanthine, B, vindoline and C, ajmalicine.

Analyzing the evolution of coronaridine content in leaves from seedlings at 2–9 weeks after sowing revealed the peak content at 580 µg g^−1^ F.W. at 3 weeks after sowing. Subsequently, as the number of days after sowing increased, coronaridine content gradually declined, reaching approximately 40 mg g^−1^ F.W. in plants at 9 weeks after sowing (Figure 9). Typically, flower buds emerge around the 9-week mark, and with further growth leading to pod formation, coronaridine becomes challenging to detect. This underscores the significant changes in coronaridine content dependent on the growth cycle. In investigating other alkaloids in *C. roseus*—catharanthine, vindoline, and ajmalicine—it was found that catharanthine (Figure 10A) and vindoline (Figure 10B) remained relatively constant irrespective of the growing season. Conversely, ajmalicine closely mirrored the change in coronaridine content, being abundant in young plants and diminishing as they matured. However, even as growth progressed, trace amounts of ajmalicine persisted and did not go unnoticed (Figure 10C).

## Discussion

This study aims to provide detailed insights into the accumulation of coronaridine in *C. roseus*. Referencing a report on coronaridine analysis in *T. iboga* (Farrow et al. 2019), we conducted LC-MS and LC-MS/MS analyses, discovering the presence of coronaridine in the methanol extract of *C. roseus* seedling leaves. Additionally, Farrow et al. (2019) noted the existence of (−) and (+) isomers of coronaridine in *T. iboga*, with *T. iboga* containing the (−) isomer. Our LC-DC analysis confirmed that coronaridine in *C. roseus* is of the (−) type, same to that in *T. iboga*. These findings underscore the biosynthesis of coronaridine in *C. roseus*, positioning it as a precursor within the metabolic pathway of iboga alkaloids.

It has been reported that the levels of the alkaloids catharanthine, vindoline, and vinblastine in the leaves of *C. roseus* are increased by high temperatures (Guo et al. 2007) and suppressed by low temperatures (Dutta et al. 2007, 2013). Therefore, when we compared the coronaridine content of *C. roseus* grown at 25°C and at 35°C, we found that the coronaridine content was significantly higher when grown at 35°C than when grown at 25°C. Furthermore, when we analyzed changes in the amount of coronaridine accumulated depending on the growth stage, we found that the content of coronaridine contained per leaf weight was high in young plants, with the accumulated amount peaking in the 3rd to 4th weeks after sowing. During the subsequent growth period, the coronaridine content per plant remained constant, suggesting that the coronaridine content per leaf weight decreased as the plant grew. Until now, there have been few reports of the presence of coronaridine in *C. roseus* plants because mature plants have been used as research materials.

This study is the first to show that *C. roseus* contains coronaridine and that it is the same isomer as coronaridine found in *T. iboga*. It is expected that iboga alkaloids can be synthesized semisynthetically using coronaridine found in *C. roseus*.

## Abbreviations

CD: circular dichroism spectrum
GC-MS: gas chromatography-mass spectrometry
LC-MS: liquid chromatography-mass spectrometry
RT: retention time
